# The Long-Term Effectiveness of Psychoeducation for Bipolar Disorders in Mental Health Services. A 4-Year Follow-Up Study

**DOI:** 10.3389/fpsyt.2019.00873

**Published:** 2019-11-26

**Authors:** Chiara Buizza, Valentina Candini, Clarissa Ferrari, Alberto Ghilardi, Francesco Maria Saviotti, Cesare Turrina, Gianluigi Nobili, Margherita Sabaudo, Giovanni de Girolamo

**Affiliations:** ^1^Department of Clinical and Experimental Sciences, University of Brescia, Brescia, Italy; ^2^Psychiatric Epidemiology and Evaluation Unit, IRCCS Istituto Centro San Giovanni di Dio Fatebenefratelli, Brescia, Italy; ^3^Service of Statistics, IRCCS Istituto Centro San Giovanni di Dio Fatebenefratelli, Brescia, Italy; ^4^Department of Mental Health, ASST Garda, Brescia, Italy; ^5^Department of Mental Health, ASST Spedali Civili, Brescia, Italy

**Keywords:** psychoeducation, effectiveness, follow-up, hospitalizations, integrated treatment, bipolar disorder

## Abstract

**Aims:** The aims of the present study were to assess: the effectiveness of psychoeducation in mental health service (MHSs) in terms of time to first hospitalization during 4-year follow-up; the number and the days of hospitalizations, and the number of people hospitalized at 4-year follow-up; and variables associated with better outcome in BD patients.

**Methods:** This is a controlled study involving an experimental group (N = 57) and a control group (N = 52). The treatment phase consists of 21 weeks, in which all participants received TAU, while the experimental group received additional psychoeducation.

**Results:** The survival analysis showed significant differences in terms of time to first hospitalization of up to 4-year follow-up: the patients in the psychoeducation group showed a longer time free from hospitalizations than the control group. Concerning the predictors of time to first hospitalization, the only factor that showed a trend to statistical significance was psychoeducation.

**Conclusions:** This is one of few studies assessing the long-term effectiveness of psychoeducation in a naturalistic setting. The data confirm that psychoeducation can impact illness course, in terms of longer time free from hospitalizations.

**Trial registration:** ISRCTN17827459

## Introduction

Bipolar disorder (BD) is a chronic and recurrent mental disorder, which often causes severe disability among people who suffer from it. Even though medication is needed, the role of psychosocial factors both in the onset and in the progression of BD has become progressively evident and has led to the development of several psychosocial approaches as adjunctive treatment to pharmacological therapies. Among the several psychological treatments, psychoeducation has shown its efficacy, so that recent reviews of evidence-based guidelines for the clinical management of BD state that “all patients with BD should be offered group or individual psychoeducation” (1, 2). Many studies have confirmed that psychoeducation is effective in helping people with BD detect early signs and implement behavioral measures to prevent full-blown episodes, which are frequently associated with high morbidity and more hospitalizations (3–7). Moreover, Chatterton and colleagues (8) in their meta-analysis showed that psychoeducation is very effective to improve medication adherence. A current review, aimed to assess the literature on the efficacy of several types of psychoeducation (individual, group, family, internet-based), showed that group and family psychoeducation are the most efficacious; in contrast, the individual and internet psychoeducation need further study (9).

Although to date the benefits of group psychoeducation in the management of BD are well known, the evidence that the positive effects of psychoeducation persist over time is still weak; moreover, there are few studies about effectiveness of psychoeducation provided in ordinary Mental Health Services, as it was investigated in the present study. Meyer and Hautzinger (10) have shown, for example, that there were no differences in relapse between treatment conditions over 2-year follow-up, pointing out that some shared aspects, such as information or regular mood monitoring, might explicate the effects of psychological treatment for BD.

In a previous study, we evaluated the effectiveness of psychoeducation at 1-year follow-up (11) comparing two groups: one group attended psychoeducation and one group was in a waiting list (control group).

The results showed that the number of patients hospitalized during the 1-year follow-up, the mean number of hospitalizations per patient, and the mean number of hospitalization days were significantly lower for psychoeducation patients.

In this study, we want to evaluate the outcomes of psychoeducation at 4-year follow-up, in order to assess the long-term effectiveness of psychoeducation over time. Furthermore, we wanted to see if there are variables that can predict who will better respond to psychoeducation. In fact, there are still two key questions that need to be addressed: how to predict who will most benefit from psychoeducation, and therefore to which patients to recommend it.

## Materials and Methods

### Study Design

This controlled study involved two groups of outpatients: patients in the experimental group received treatment as usual (TAU), consisting of one monthly visit with the treating psychiatrist and pharmacological treatment specific for BD, and additional psychoeducation according to Colom and Vieta's model (12); patients in the control group received only TAU. During the 4-year follow-up, all participants continued to receive TAU; the experimental group did not receive boosting sessions of psychoeducation.

### Participants

One hundred and twenty-seven outpatients with BD, aged 18–65 years, were involved in this study. Eighteen were excluded: 13 not meeting inclusion criteria and 5 declined to participate.

The study is a pragmatic trial conducted under routine conditions and so randomization was not possible. Two DMHs (DMH-A and DMH-B) located in Brescia, a northern Italian town, were involved: patients were selected and evaluated at both DMHs. Psychoeducation was implemented only at DMH-A; DMH-B, where psychoeducation was never implemented for organizational reasons, represented the control group (11). Furthermore, during the 4-year follow-up, 13 (28.8%) patients dropped out of treatment at DMH-B; since the size of the control sample became too small for a proper comparison, we randomly selected 20 additional patients meeting inclusion criteria from the DMH-B electronic registry to be added to the original control sample; these patients underwent the same evaluation of the original sample (see below).

Inclusion criteria were diagnosis of BD type I or II; being euthymic for at least 3 months; information about illness course during ≥18 months prior to start of psychoeducation (collected from the medical record and from the psychiatrist); willingness to continue current medication; and written informed consent to participate in group psychoeducation. Exclusion criteria included all DSM-IV Axis I disorders; mental retardation (IQ <70); current substance use such as alcohol, cannabis, cocaine, etc. (except for tobacco smoking); organic brain damage, or deafness. Patients undergoing any structured form of psychological treatment were also excluded.

The study was approved by Ethical Committee of the Saint John of God, Fatebenefratelli of Brescia (N° 96/2009/I). All procedures performed in this study were in accordance with the 1964 Helsinki declaration and its later amendments or comparable ethical standards. All participants have written/wrote informed consent to participate in group psychoeducation.

### Psychoeducation Group

Group psychoeducation was performed according to Colom and Vieta's model, consisting of 21 weekly sessions of 90 min, each aiming at improving four main areas: illness awareness, treatment adherence, early detection of warning signs of a probable episode, and lifestyle regularity (12). The psychoeducation was delivered in groups of 8–12 participants, conducted by two clinical psychologists, who had previously attended a training psychoeducation course directly held by Francesc Colom. Patients missing more than five sessions were excluded from the group to avoid the potential for insufficient treatment “dosages” in cases producing nil results.

### Standardized Assessment

Before inclusion in the study, all patients in both groups were assessed through the following tools: the Structured Clinical Interview for DSM-IV Axis I (SCID-I) to confirm BD diagnosis; the Young Mania Rating Scale (YMRS), and the Hamilton Rating Scale for Depression (HAM-D-17) in order to assess euthymia. The cut-offs of HAM-D-17 < 8 and YMRS <6 were identified by previous studies (13). There were no differences between the two groups in the mean level of mood symptoms at the baseline. The average of the HAM-D-17 total score was 4.64 (SD = 3.5) for the psychoeducation group and 5.14 (SD = 3.1) for the control group (U = 817: p = 0.344). The average of the YMRS total score was 3.7 (SD = 3.3) for the psychoeducation group and 3.8 (SD = 3.3) for the control group (U = 1207: p = 0.830).

Personality disorders were detected through the clinician's diagnosis in medical records. Finally, socio-demographic, clinical, and treatment-related information were collected through the Patient Schedule.

### Main Outcome Measurements

The main aims of this study were: (a) to assess the number and the days of hospitalizations, and the number of people hospitalized at 4-year follow-up; (b) to assess the effectiveness of psychoeducation in ordinary mental health services in terms of time to first hospitalization during the 4-year follow-up; and (c) to identify possible variables associated with better outcome over time with BD patients who attended group psychoeducation, and to understand who benefitted from psychoeducation. All data concerning hospitalization were collected from the Lombardy Region's electronic Mental Health Information System, which saves mandatory information concerning all hospital admissions to all General Hospital Psychiatric Units (GHPUs). As a result, we ensured that all information concerning hospitalization was accurate and reliable.

### Statistical Analysis

Sample characteristics were provided in terms of descriptive statistics including frequencies, percentages (for qualitative variables), and mean and standard deviation (for quantitative variables). Differences between psychoeducation and control groups were tested with a chi-square test for categorical variables and by T-test for Gaussian distributed quantitative variables (or non-parametric Mann–Whitney test for non-Gaussian variables). Normality assumption was tested with a Shapiro–Wilk test as well as Kolmogorov–Smirnov test (data not shown). All tests were two-tailed and the probability of a type I error was set at p = 0.05.

The Kaplan–Meier (KM) survival analysis was used to analyze the hospitalization-free curves at 4-year follow-up time. Differences of KM-curves between the two groups were evaluated by Log-rank test. Cox proportional-hazards regression model were performed to analyze the dependency of time to first hospitalization on predictor variables (14). All analyses were conducted according to an “intention-to-treat” model, including all patients who started but did not complete psychoeducation (drop-outs). Analysis were performed by using SPSS 23.0 and by survival package of R: A language and environmental for statistical computing (version 3.4.1).

## Results

### Patients’ Recruitment and Drop-Out

Overall, 109 euthymic outpatients were recruited: 57 patients were enrolled in the experimental group and 52 in the control group. In the psychoeducation group, 46 individuals out of 57 (80.7%) completed psychoeducation program, attending a mean of 19.3 sessions (SD = 0.9). Eleven participants (19.3%) withdrew from the group for various reasons: manic recurrence (1 patient), depressive recurrence (1 patient), mixed recurrence (2 patients), conflicting schedules (3 patients), or disagreement with the biological approach underlying the cause of BD (4 patients). In all cases, drop-out participants attended a mean of 10.4 sessions (SD = 5.3).

### Samples’ Socio-Demographic Characteristics

The samples’ characteristics at baseline are reported in **Table 1**; both groups were comparable regarding socio-demographic and clinical characteristics. Despite 20 patients added to the control group, these results show that the features of new patients did not diverge from those of the patients in the original control group; indeed the sociodemographic and clinical characteristics of the new control group did not differ significantly from those of the experimental group.

**Table 1:** Sociodemographic and clinical characteristics of study participants.

Characteristics	Psychoeducation group (*n* = 57)	Control group (*n* = 52)	Statistical test	*p-value*
Male gender, *n* (%)	27 (47.4)	27 (51.9)	*.00* *^a^*	*.94*
Mean age (*sd*)	41.5 (9.1)	41.7 (10.1)	-*.12* ^b^	*.90*
Mean education, *years* (*sd*)	11.8 (3.5)	10.6 (3.5)	*1.7* ^b^	*.07*
In employment, *n* (%)	28 (49.1)	30 (57.6)	*.80* *^a^*	*.37*
Marital status, married, *n* (%)	22 (38.6)	21 (40.3)	*.03* ^a^	*.84*
Diagnostic subtype, bipolar I, *n* (%)	55 (96.5)	49 (94.2)	*.31* ^a^	*.57*
Rapid cycling*, yes, *n* (%)	3 (5.2)	0 (0.0)	*2.7* *^a^*	*.09*
Presence of psychotic symptoms*, *n* (*%*)	51 (89.4)	39 (78.0)	*2.6* *^a^*	*.10*
Presence of attempted suicide*, *n* (%)	12 (21.0)	11 (21.1)	*.00* *^a^*	*.94*
Mean age of onset, *years* (*sd*)	29.0 (8.5)	27.8 (8.2)	*.75* ^b^	*.45*
Mean age of first contact with Mental Health Services, *years* (*sd*)	32.6 (8.7)	31.8 (9.0)	*.44* ^b^	*.65*
Number of previous hospitalizations^#^, *mean* (*sd*) Personality disorders, *n* (%)	0.4 (0.7) 10 (17.5)	0.3 (0.7) 15 (28.8)	*1.3* ^c^ 2.3^a^	*.34* *.12*

^a^Chi-squared test; df = 1; ^b^t-test; ^c^Mann–Whitney; *Lifetime history; ^#^In the 18 months before the start of psychoeducation.

### Number of People Hospitalized During the 4-Year Follow-Up

No significant differences between-group (χ2 = 3.09, p = 0.109) were observed during the 4-year follow-up: 23 (44.2%) individuals out of 52 in the control group were hospitalized and 16 (28.1%) out of 57 in the psychoeducation group.

### Number of Hospitalizations During the 4-Year Follow-Up

During the 4-year follow-up, the psychoeducation group had a mean number of 0.5 (SD = 0.97) hospital admissions versus 0.9 (SD = 1.40) observed among controls (U = 1732; p = 0.076).

### Number of Hospitalization Days During the 4-Year Follow-Up

During the 4-year follow-up, the psychoeducation group reported a mean of 9.3 (SD = 20.08) hospitalization days as compared to 16.8 (SD = 27.23) in the control group (U = 1735; p = 0.074).

### Hospital Admissions and Days of Hospitalization Among Completers During the 4-Year Follow-Up

In addition to the “intention-to-treat” analysis, including all patients who started but did not conclude psychoeducation, we assessed the effectiveness of psychoeducation among completers (89.9% of the sample). During the 4-year follow-up, no significant differences between-group in the number of people hospitalized were detected: 23 out of 52 control participants had been hospitalized versus 12 out of 46 in the completer psychoeducation group (chi-square = 3.50; p = 0.074). However, we observed a reduction in the mean number of hospitalizations in the group completing the psychoeducation program, as compared to the control group (0.3 versus 0.9; U = 14339, p = 0.043) and in mean days of hospitalization (6.9 versus 16.8; U = 1441, p = 0.042).

### Time to Hospitalization At 4-Year Follow-Up

**Figure 1:**
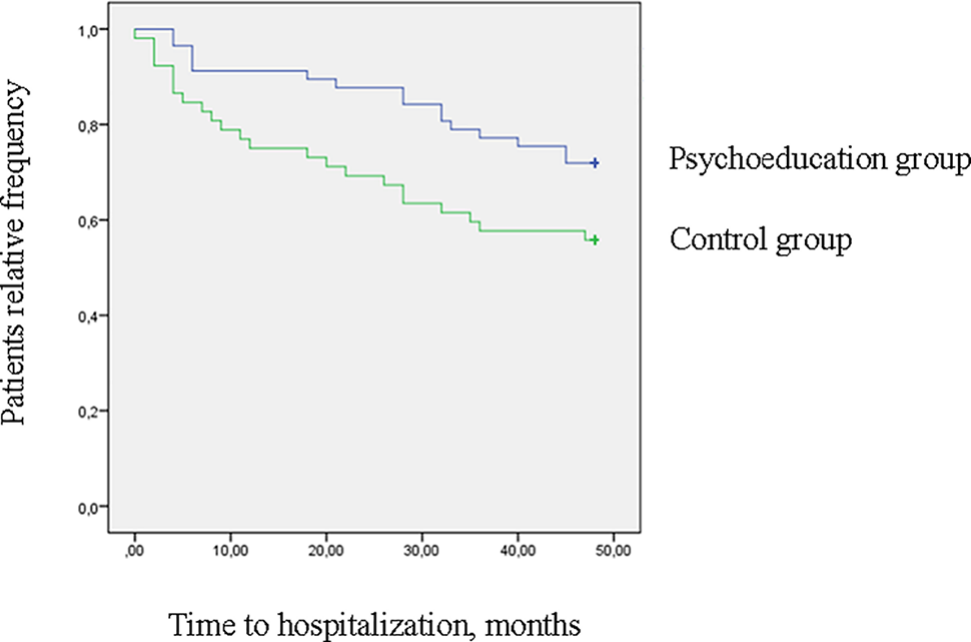
Survival curves for hospitalization at 4-year follow-up (log rank = 3.9, *p* = .047) (intention to treat analysis).

**Figure 2:**
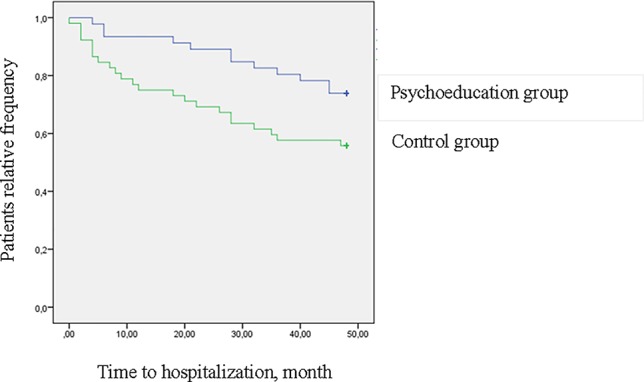
Survival curves for hospitalization at 4-year follow-up (log rank = 4.3, *p* = .037) (completers).

**Figure 1** shows the survival analysis for patients' time to first hospitalization. The two groups' event curves differed significantly in terms of time to hospitalization in the ITT analysis (log rank = 3.9, *p* < .047). The completers' event curves are shown in **Figure 2**. The between-group differences were also significant in this instance (log rank = 4.3, *p* < .037). In both cases, the patients in the psychoeducation group showed a longer time free from hospitalizations than the control group.

**Figure 3:**
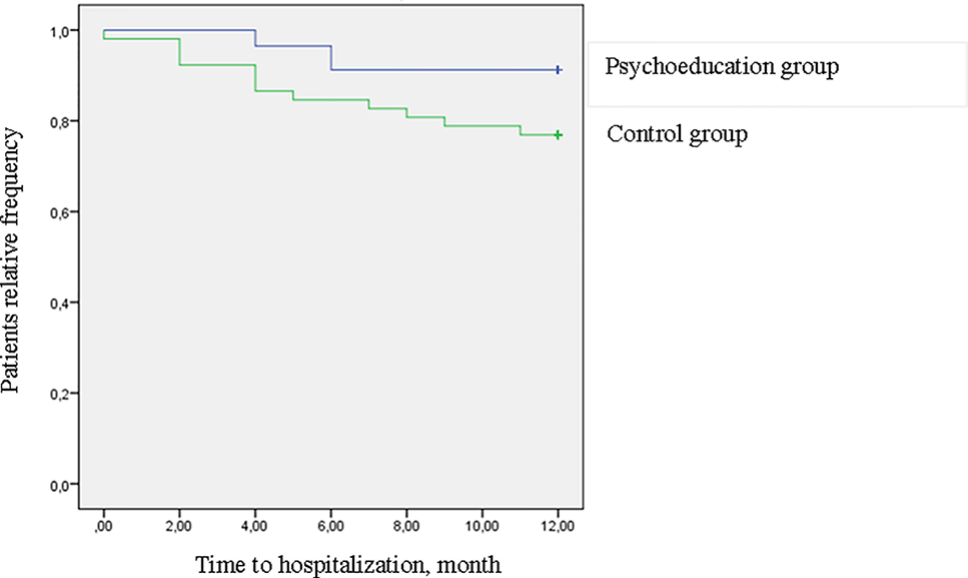
Survival curves for hospitalization at 1-year follow-up (log rank = 4.34, *p* = .037) (intention to treat analysis).

**Figure 4:**
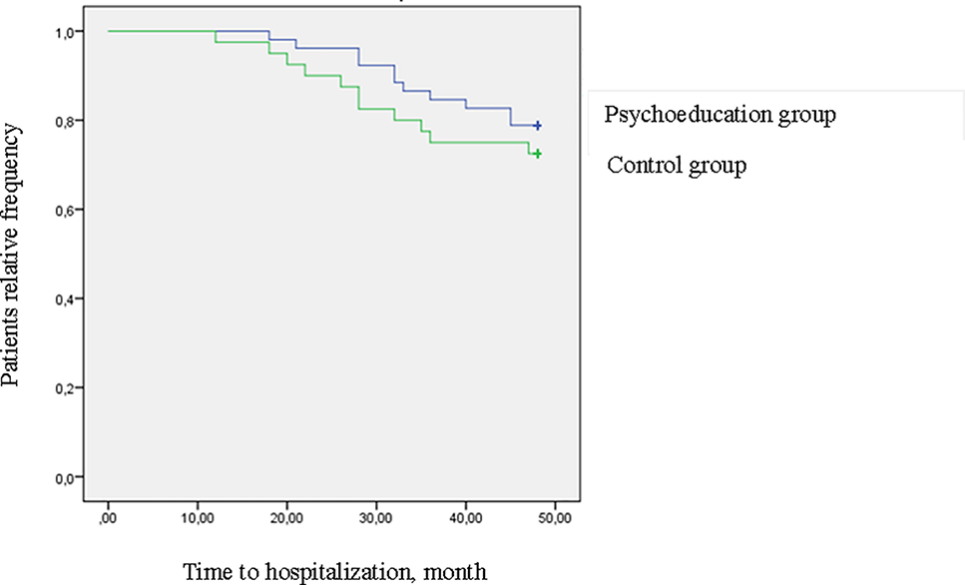
Survival curves for hospitalization at 2–4 years follow-up (log rank = 0.65 *p* = .419) (intention to treat analysis).

However, it should be noticed that the difference between the two groups during 4-year follow-up is mainly due to the difference found at the 1-year follow-up. In fact, in the following years (2–4 years), the differences between the two groups tend to decrease: this finding is well noticeable looking at **Figures 3** and **4**.

### Predictors of Time to First Hospitalization

No socio-demographic or clinical variables were associated with a shorter/longer time to first hospitalization [with hazard ratios (HRs) close to 1; **Table 2**]. The only factor that showed a trend to statistical significance with longer time to first hospitalization was “psychoeducation”: patients who attended psychoeducation had a longer time to first hospitalization than patients in the control group (HR = 0.53; 95% CI: 0.28–1.01).

**Table 2:** Predictors of time to remission. Cox proportional-hazards regression (univariate) models output (completers/[ITT]).

Predictors of remission time	Hazard Ratio HR	95% CI of HR	*p-value*
Gender, *female versus male*	0.81	0.43–1.53	*.513*
Age, *years*	0.99	0.96–1.03	*.742*
Education, *years*	1.01	0.92–1.10	*.826*
Employed, *yes versus no*	0.76	0.40–1.43	*.394*
Marital status, *yes versus no*	0.78	0.40–1.52	*.469*
Diagnostic subtype, Bipolar I, *yes versus no*	21.9	0.05–930.69	*.317*
Personality disorder, *yes versus no*	0.76	0.38–1.54	*.454*
Age of onset, *years*	1.01	0.98–1.05	*.504*
Duration of illness, *years*	0.98	0.94–1.02	*.324*
Study group, *psychoeducation versus control*	0.53	0.28–1.01	*.052*

## Discussion

To our knowledge, this is one of the few studies that has tested the long-term effectiveness of psychological interventions for BD with a long follow-up (13, 15). Furthermore, it is important to emphasize that in this study, psychoeducation was provided in a naturalistic setting of mental health services’ daily activities.

Our study confirms that group psychoeducation conferred a long-lasting prophylactic effect, in terms of longer time free from hospitalizations, as shown in the survival curves.

However, further analysis shows that differences between the two groups during the 4 years decrease significantly after the first-year follow-up. This result suggests that it might be useful to implement boosting sessions after the end of the psychoeducation, in order to prolong the period of time free from hospitalization. The boosting sessions may help patients remember protective lifestyles and risk factors for BD, and encourage a rigorous monitoring of prodromes, in order to prevent relapse.

It is interesting to observe that the number of hospitalizations and days of hospitalization among patients who completed psychoeducation was also significantly lower than in the control group. This indicates that patients who completed psychoeducation were more able to recognize prodromes early, avoiding the most severe recurrences, which require hospitalization: even when hospitalization was necessary, it was shorter. In contrast, according to ITT analysis, the differences between the groups were not statistically significant, probably due to the insufficient “dose”of treatment among non-completers. In any event, even the ITT analysis shows that those who started psychoeducation had a number of hospitalizations and days of hospitalization lower than the control group. Although this difference did not reach statistical significance, it may be considered clinically relevant, in particular with regard to the number of days of hospitalization, which in the psychoeducation group was almost half of the control group. As a comparison, it is very doubtful that any mood stabilizer may confer any degree of protection from recurrences during 4 years following the discontinuation of medication.

Our results showed how this time-limited single intervention brought about a major improvement in BD outcome over the long term (in our case 4 years). Therefore, group psychoeducation can enhance behavioral and lifestyle changes that seem to be maintained over time, although some patients might need boosting sessions, as other studies suggested (13, 16, 17).

The positive effects of psychoeducation would seem possible because this type of psychosocial treatment cannot be considered simple information. Psychoeducation produces a process of awareness on illness that is necessary for an effective management of BD (13). Indeed, psychoeducation includes educational and psychosocial targets that need the use of educational techniques to promote a long-lasting behavioral change in BD patients. For this reason, it is reasonable to assume that its effects are maintained over time. This assumption is confirmed by a recent meta-analysis showing that the greater improvements in mania symptoms and in social functioning at long-term follow-up relative to the short term may be indicative of increased effectiveness of psychoeducation over time (8).

### Impacts of Psychoeducation

Psychoeducation is considered very important for people suffering from BD and their relatives (18), both due to its primary efficacy for recurrence prevention and its secondary benefit associated with improved perceived social support, better awareness of BD, better attitude towards drugs, and access to services. This has been demonstrated in a recent qualitative research on patients' subjective experiences of a group psychoeducation intervention for people with BD, aimed at understanding the feasibility, acceptability, and impact of psychoeducation (19). It is possible that these secondary outcomes are determined by certain informal aspects of group setting, e.g., patients who benefited from learning from others, feeling less socially isolated. Meeting other people with BD normalized their illness experience, delivered a feeling of community. All these aspects contribute to the most important goal in the treatment of every patient with a chronic disorder (such as BD), i.e., the improvement of the perceived quality of life.

Moreover, thanks to psychoeducation, patients become more knowledgeable about medication and treatment options, improving treatment adherence. In fact, they may become more aware of the importance of drug treatment to prevent relapses. This leads them to take medication more regularly (8). Other important effects of psychoeducation include promotion of a more regular lifestyle and the early detection of prodromal signs, fundamental factors to improve the course and prognosis of BD (13, 20–22).

Finally, the greater mood stabilization produced by psychoeducation is a significant achievement in view of recent discoveries about neuroprogression in BD. Through the reduction of recurrences, psychoeducation could contrast the neuroprogression of BD, reducing brain changes and cognitive impairment (23).

### Clinical Implications: Who Benefits From Psychoeducation?

Recent studies suggested the usefulness of psychoeducation for relapse prevention only in a selected subgroup of early stage patients (24–27). Although it seems logical that psychoeducation should be offered to patients as soon as possible, our results suggest that this intervention can have a wider range of action. In our study, important variables usually associated with a worse course of BD, such as psychotic symptoms, suicide attempts, Axis II comorbidity, and early age of onset, did not produce any significant differences on the effectiveness of psychoeducation. Moreover, it is very important to consider that the secondary effects of psychoeducation above mentioned, which go beyond the prevention of relapses, produce a significant improvement in the patients' quality of life (28), probably due to a higher level of self-efficacy (29).

From these considerations, we suggest that psychoeducation should be offered to all patients with BD and not only those at an early stage of the disease. As stated in the recent evidence-based guidelines for the clinical management of BD (revised third edition recommendations from the British Association for Psychopharmacology), psychoeducation should always be the preferred psychological intervention for people suffering from BD (30).

### Limitations

This study has some limitations. The most important is its limited generalizability, caused by lack of randomization and by the small number of participants involved. Moreover, the primary outcome of this study were recurrences with hospitalization, but no information was available on mood instability like hypomanic or moderate depressive that did not require hospitalization, but which are equally important from a clinical point of view. Moreover, as the participants were not followed up, but rather their hospitalization data were used, it was also not possible to know what other psychological interventions they may have done over the 4-year period. Furthermore, most patients suffered by BD I, so we cannot be sure that similar results in terms of psychoeducation effectiveness can be also extended to patients with BD II. Finally, no data on the polarity of recurrences were also available, as well as on possible life events, which may increase the likelihood of affective episodes in BD (31).

## Conclusions

The results of this study show that patients who participated in psychoeducation had a longer time to first hospitalization, after 4 years, compared to patients who received TAU only. However, since the effectiveness of psychoeducation tends to decrease over time, it may be useful to add boosting sessions after the end of psychoeducation in order to prolong the period of time free from hospitalization.

Moreover, this study shows that there were not socio-demographic or clinical variables associated with time to first hospitalization, but the only factor related to a longer time free from hospitalizations was the attendance to psychoeducation.

These data confirm that combining pharmacological plus an evidence-based adjunctive psychosocial intervention, such as group psychoeducation, is currently the most effective way to improve BD outcomes. Although many studies have stated that psychoeducation should be applied as early as possible, our study shows that psychoeducation can have a wider range of action and be also useful for patients with a longer history of illness. For this reason, psychoeducation should be offered to all patients with BD in Mental Health Services.

## Data Availability Statement

The datasets generated for this study are available on request to the corresponding author.

## Ethics Statement

The studies involving human participants were reviewed and approved by Ethical Committee of the Saint John of God, Fatebenefratelli of Brescia (N° 96/2009/I). The patients/participants provided their written informed consent to participate in this study.

## Author Contributions

CB has managed the literature searches, designed the study, conducted the psychoeducation groups, wrote the protocol and the manuscript, and managed the analysis. VC has designed the study, conducted the psychoeducation groups, and wrote the protocol and the manuscript. CF has performed data analysis, wrote the statistical analysis section and contributed to the writing of results, and gave her final approval of the version to be published. AG managed the literature search, wrote the protocol and the manuscript, and gave his final approval of the version to be published. FMS has selected patients and helped organize the study at the Desenzano DMH. He was involved in manuscript revision and gave his final approval of the current version. CT has selected patients and helped organize the study at the Brescia DMH. He was involved in manuscript revision and gave his final approval of the current version. GN has selected patients and helped organize the study at the Desenzano DMH. He was involved in manuscript revision and gave his final approval of the current version. MS has selected patients and helped organize the study at the Desenzano DMH. He was involved in manuscript revision and gave his final approval of the current version. GDG started the project and applied for funding, managed the literature search, designed the study, wrote the protocol and the manuscript, coordinated the study, and gave his final approval of the current version. All authors read and approved the final manuscript.

## Funding

This project was funded by the Lombardy Region ‘Programmi Innovativi per la Salute Mentale’ (TR15).

## Conflict of Interest

The authors declare that the research was conducted in the absence of any commercial or financial relationships that could be construed as a potential conflict of interest.

## References

[1] Connolly KR Thase ME The clinical management of bipolar disorder: a review of evidence-based guidelines. Prim Care Companion CNS Disord (2011) 13:4. 10.4088/PCC.10r01097 PMC3219517 22132354

[2] Podawiltz A A review of current bipolar disorder treatment guidelines. J Clin Psychiatry (2012) 73(3):e12. 10.4088/JCP.10060tx2cc 22490265

[3] Swartz HA Swanson J Psychotherapy for bipolar disorder in adults: a review of the evidence. Focus (Am Psychiatr Publ) (2014) 12(3):251–66. 10.1176/appi.focus.12.3.251 PMC4536930 26279641

[4] Bond K Anderson IM Psychoeducation for relapse prevention in bipolar disorder: a systematic review of efficacy in randomized controlled trials. Bipolar Disord (2015) 17:349–62. 10.1111/bdi.12287 25594775

[5] Miziou S Tsitsipa E Moysidou S Karavelas V Dimelis D Polyzoidou V Psychosocial treatment and interventions for bipolar disorder: a systematic review. Ann Gen Psychiatry (2015) 14:19. 10.1186/s12991-015-0057-z 26155299 PMC4493813

[6] Salcedo S Gold AK Sheikh S Marcus PH Nierenberg AA Deckersbach T Empirically supported psychosocial interventions for bipolar disorder: current state of the research. J Affect Disord (2016) 201:203–14. 10.1016/j.jad.2016.05.018 27243619

[7] Oud M Mayo-Wilson E Braidwood R Schulte P Jones SH Morriss R Psychological interventions for adults with bipolar disorder: systematic review and meta-analysis. Br J Psychiatry (2016) 208:213–22. 10.1192/bjp.bp.114.157123 26932483

[8] Chatterton ML Stockings E Berk M Barendregt JJ Carter R Mihalopoulos C Psychosocial therapies for the adjunctive treatment of bipolar disorder in adults: network meta-analysis. Br J Psychiatry (2017) 210(5):333–41. 10.1192/bjp.bp.116.195321 28209591

[9] Soo SA Zhang ZW Khong SJ Low JEW Thambyrajah VS Alhabsyi SHBT Randomized controlled trials of psychoeducation modalities in the management of bipolar disorder: a systematic review. J Clin Psychiatry (2018) 79(3):pii:. 10.4088/JCP.17r11750 29727072

[10] Meyer TD Hautzinger M Cognitive behaviour therapy and supportive therapy for bipolar disorders: relapse rates for treatment period and 2-year follow-up. Psychol Med (2012) 42:1429–39. 10.1017/S0033291711002522 22099722

[11] Candini V Buizza C Ferrari C Caldera MT Ermentini R Ghilardi A Is structured group psychoeducation for bipolar patients effective in ordinary mental health services? A controlled trial in Italy. J Affect Disord (2013) 151(1):149–55. 10.1016/j.jad.2013.05.069 23816448

[12] Colom F Vieta E Manuale di psicoeducazione per il disturbo bipolare (2006). Giovanni Fioriti Editore: Roma (2006).

[13] Colom F Vieta E Sánchez-Moreno J Palomino-Otiniano R Reinares M Goikolea JM Group psychoeducation for stabilised bipolar disorders: 5-year outcome of a randomised clinical trial. Br J Psychiatry (2009) 194:260–5. 10.1192/bjp.bp.107.040485 19252157

[14] Laine T Reyes EM Tutorial: survival estimation for Cox Regression Models with time-varying coefficients using SAS, and R. J Stat Software (2014) 61:1–23. 10.18637/jss.v061.c01

[15] González Isasi A Echeburúa E Limiñana JM González-Pinto A Psychoeducation and cognitive-behavioral therapy for patients with refractory bipolar disorder: a 5-year controlled clinical trial. Eur Psychiat (2014) 29(3):134–41. 10.1016/j.eurpsy.2012.11.002 23276524

[16] Lam D Donaldson C Brown Y Malliaris Y Burden and marital and sexual satisfaction in the partners of bipolar patients. Bipolar Disord (2005) 7(5):431–40. 10.1111/j.1399-5618.2005.00240.x 16176436

[17] Ball JR Mitchell PB Corry JC Skillecorn A Smith M Malhi GS A randomized controlled trial of cognitive therapy for bipolar disorder: focus on long-term change. J Clin Psychiatry (2006) 67(2):277–86. 10.4088/jcp.v67n0215 16566624

[18] Gex-Fabry M Cuénoud S Stauffer-Corminboeuf MJ Aillon N Perroud N Aubry JM Group psychoeducation for relatives of persons with bipolar disorder: perceived benefits for participants and patients. J Nerv Ment Dis (2015) 203(9):730–4. 10.1097/NMD.0000000000000355 26313039

[19] Poole R Smith D Simpson S Patients' perspectives of the feasibility, acceptability and impact of a group-based psychoeducation programme for bipolar disorder: a qualitative analysis. BMC Psychiatry (2015) 15:184. 10.1186/s12888-015-0556-0 26231750 PMC4522139

[20] Perry A Tarrier N Morriss R McCarthy E Limb K Randomised controlled trial of efficacy of teaching patients with bipolar disorder to identify early symptoms of relapse and obtain treatment. BMJ (1999) 318(7177):149–53. 10.1136/bmj.318.7177.149 PMC27688 9888904

[21] Colom F Vieta E Sánchez-Moreno J Martínez-Arán A Reinares M Goikolea JM Stabilizing the stabilizer: group psychoeducation enhances the stability of serum lithium levels. Bipolar Disord (2005) 7 Suppl 5:32–6. do 10.1111/j.1399-5618.2005.00249.x 16225558

[22] Frank E Kupfer DJ Thase ME Mallinger AG Swartz HA Fagiolini AM Two-year outcomes for interpersonal and social rhythm therapy in individuals with bipolar I disorder. Arch Gen Psychiatry (2005) 62(9):996–1004. 10.1001/archpsyc.62.9.996 16143731

[23] Passos IC Mwangi B Vieta E Berk M Kapczinski F Areas of controversy in neuroprogression in bipolar disorder. Acta Psychiatr Scand (2016) 134(2):91–103. 10.1111/acps.12581 27097559

[24] Miller IW Solomon DA Ryan CE Keitner GI Does adjunctive family therapy enhance recovery from bipolar I mood episodes? J Affect Disord (2004) 82(3):431–6. . 10.1016/j.jad.2004.01.010. 15555694

[25] Scott J Colom F Vieta E A meta-analysis of relapse rates with adjunctive psychological therapies compared to usual psychiatric treatment for bipolar disorders. Int J Neuropsychopharmacol (2007) 10(1):123–9. 10.1017/S1461145706006900 16787554

[26] de Barros Pellegrinelli K de O Costa L Silval KI Dias VV Roso MC Bandeira M Efficacy of psychoeducation on symptomatic and functional recovery in bipolar disorder. Acta Psychiatr Scand (2013) 127(2):153–8. 10.1111/acps.12007 22943487

[27] Morriss R Lobban F Riste L Davies L Holland F Long R Clinical effectiveness and acceptability of structured group psychoeducation versus optimised unstructured peer support for patients with remitted bipolar disorder (PARADES): a pragmatic, multicentre, observer-blind, randomised controlled superiority trial. Lancet Psychiatry (2016) 3(11):1029–38. 10.1016/S2215-0366(16)30302-9 27688021

[28] Faridhosseini F Baniasadi M Fayyazi Bordbar MR Pourgholami M Ahrari S Asgharipour N Effectiveness of psychoeducational group training on quality of life and recurrence of patients with bipolar disorder. Iran J Psychiatry (2017) 12(1):21–8. PMC5425348 28496498

[29] Abraham K Miller CJ Birgenheir DG Lai Z Kilbourne A Self-Efficacy and Quality of Life Among People With Bipolar Disorder. J Nerv Ment Dis (2014) 202(8):583–8. 10.1097/NMD.0000000000000165 PMC4133989 25010107

[30] Goodwin GM Haddad PM Ferrier IN Aronson JK Barnes TRH Cipriani A Evidence-based guidelines for treating bipolar disorder: Revised third edition recommendations from the British Association for Psychopharmacology. J Psychopharmacol (2016) 30(6):495–553. 10.1177/0269881116636545 26979387 PMC4922419

[31] Simhandl C Radua J König B Amann BL The prevalence and effect of life events in 222 bipolar I and II patients: a prospective, naturalistic 4 year follow-up study. J Affect Disord (2015) 170:166–71. 10.1016/j.jad.2014.08.043 25240845

